# Increasing myosin light chain 3f (MLC3f) protects against a decline in contractile velocity

**DOI:** 10.1371/journal.pone.0214982

**Published:** 2019-04-09

**Authors:** Jong-Hee Kim, Ted G. Graber, Haiming Liu, Atsushi Asakura, LaDora V. Thompson

**Affiliations:** 1 Department of Physical Education, Hanyang University, Seoul, South Korea; 2 Department of Nutrition and Metabolism, University of Texas Medical Branch, Galveston, TX, United States of America; 3 Department of Medicine, University of Washington, Seattle, Washington, United States of America; 4 Department of Neurology, University of Minnesota Medical School, Minneapolis, MN, United States of America; 5 Department of Physical Therapy and Athletic Training, Boston University, Boston, MA, United States of America; University of Louisville School of Medicine, UNITED STATES

## Abstract

Disuse induces adaptations in skeletal muscle, which lead to muscle deterioration. Hindlimb-unloading (HU) is a well-established model to investigate cellular mechanisms responsible for disuse-induced skeletal muscle dysfunction. In myosin heavy chain (MHC) type IIB fibers HU induces a reduction in contraction speed (Vo) and a reduction in the relative myosin light chain 3f (MLC3f) protein content compared with myosin light chain 1f (MLC1f) protein. This study tested the hypothesis that increasing the relative MLC3f protein content via rAd-MLC3f vector delivery would attenuate the HU-induced decline in Vo in single MHC type IIB fibers. Fischer-344 rats were randomly assigned to one of three groups: control, HU for 7 days, and HU for 7 days plus rAd-MLC3f. The semimembranosus muscles were injected with rAd-MLC3f (3.75 x 10^11^–5 x 10^11^ ifu/ml) at four days after the initiation of HU. In single MHC type IIB fibers the relative MLC3f content decreased by 25% (12.00±0.60% to 9.06±0.66%) and Vo was reduced by 29% (3.22±0.14fl/s vs. 2.27±0.08fl/s) with HU compared to the control group. The rAd-MLC3f injection resulted in an increase in the relative MLC3f content (12.26±1.19%) and a concomitant increase in Vo (2.90±0.15fl/s) of MHC type IIB fibers. A positive relationship was observed between the percent of MLC3f content and Vo. Maximal isometric force and specific tension were reduced with HU by 49% (741.45±44.24μN to 379.09±23.77μN) and 33% (97.58±4.25kN/m^2^ to 65.05±2.71kN/m^2^), respectively compared to the control group. The rAd-MLC3f injection did not change the HU-induced decline in force or specific tension. Collectively, these results indicate that rAd-MLC3f injection rescues hindlimb unloading-induced decline in Vo in MHC type IIB single muscle fibers.

## Introduction

Contractile velocity (Vo, unloaded shortening velocity) in single skeletal muscle fibers is dependent on the type of myosin heavy chain (MHCI, MHCIIA, MHCIIB) and the composition of the essential myosin light chain isoforms (MLC1f, MLC3f) [1,2]. The MHC isoform influences contractile velocity by its ability to hydrolyze ATP [3,4], whereas the essential myosin light chains fine-tune the speed of contraction. This fine-tuning role is demonstrated in single fiber contractility experimentation in which single MHC type IIB fibers with more relative MLC3f content contract faster compared to fibers with more relative MLC1f content, in which both essential MLC isoform genes are generated from a single gene locus via alternative and different modes of splicing [2,5]. This fine-tuning control of contractile velocity by the essential myosin light chains is linked to the interaction of the N-terminus of the MLC with actin during the myosin-actin cross-bridge cycle [6].

In hindlimb unloading (HU) investigations the soleus muscle has been extensively evaluated and the results show atrophy, weakness, and an increase in Vo in MHC type I fibers [7,8]. A selective loss in actin is one of the reported underlying mechanisms responsible for the increased Vo in MHC type I fibers. The increase in velocity occurs because the loss in actin increases the lateral distance (lattice spacing) between actin and myosin, allowing myosin to detach from actin earlier during cross-bridge cycles, resulting in a reduction in the internal drag. Another potential contributing mechanism lies in changes in the MLC isoform composition within individual fibers. In particular, HU also results in changes in the MLC isoform composition of the soleus, a slow-to-fast transition, with significant increases in relative MLC3f content [9]. The increase in the relative MLC3f protein content favors a faster contraction velocity [6].

In contrast to the soleus muscle, HU reduces Vo in MHC type IIB fibers from the hamstring (i.e., semimembranosus, SM) [10] and calf (i.e., gastrocnemius) [11] muscles. The mechanisms responsible for the decreased Vo seem to be less associated with changes in ATP hydrolysis or lattice spacing [10]. Instead, the essential myosin light chains, MLC1f and MLC3f as regulators of velocity, have been suggested to be involved with HU-induced decline in Vo [10]. In fact, the relative MLC3f protein content is reduced and the relative MLC1f protein content is increased with HU [10] as Vo decreases in MHC type IIB fibers from the SM muscle.

The impact of the essential myosin light chains on contractile velocity is observed in other conditions [12,13]. For example, aging is associated with a reduction in velocity in MHC type IIB fibers [12]. The decline in velocity is associated with changes in MLC isoform expression, specifically with a decline in MLC3f content in MHC type IIB fiber [12]. At the level of the whole muscle, *in vitro* contractile function of the murine extensor digitorum longus (consisting mainly of MHC type IIB) shows declining velocity with age, with a corresponding decrease in MLC3f relative content [12]. More importantly, increasing MLC3f content via recombinant adenovirus (rAd)-MLC3f DNA injection rescues the age-related decline in contractile velocity [12]. This is an important aspect because it is plausible that increasing MLC3f protein content would attenuate the deterioration in velocity following non-weight bearing conditions (e.g., hindlimb unloading) in MHC type IIB fibers. Preventing the disadvantages associated with non-weighting bearing conditions has potential to shorten the recovery period, facilitate bone and muscle health, and improve quality of life.

In the current study, our primary goal was to prevent HU-induced decline in contractile velocity in MHC type IIB fibers. We tested the hypothesis that increasing MLC3f protein content via rAd-MLC3f delivery would attenuate HU-induced decline in Vo in MHC type IIB fibers.

## Materials and methods

### The rAd-MLC3f DNA construction

To construct the recombinant adenovirus (rAd) rat MLC3f expression vector, we followed the protocol as previously described [12]. First, total RNA from the rat SM muscle was extracted using TRIZOL reagent (Invitrogen, Carlsbad, CA). The cDNA was synthesized using the Transcriptor first strand cDNA synthesis kit (Roche Applied Science, Indianapolis). Rat MLC3f cDNA (forward primer: 5’-TCT CCA GTC CCG CTG CTG TTT TGC-3’; reverse primer: 5’-ATT TGT GGG ATT GGT GCC CAG AGC-3’) was amplified by RT-PCR with Pfx Polymerase (Invitrogen) and first cloned into the pCRII-TOPO vector by TOPO TA cloning (Invitrogen). Plasmid DNAs were purified with QIAprep Miniprep kit (QIAGEN, Valencia, CA) and confirmed by DNA sequence analysis (Biomedical Genomics Center, University of Minnesota). After MLC3f cDNA was excised from pCRII-MLC3f with BamHI and Xhol, MLC3f cDNA was cloned into BamHI / Sall site of pDNR-CMV (Clontech). The MLC3f cDNA vector was transfected into primary myoblast using Lipofectamine 2000 (Invitrogen). MLC3f expression was verified using MLC1f/3f antibody (F310, Developmental Study Hybridoma Bank, Iowa). The rAd-MLC3f was created with the Adeno-X ViraTrak Expression System 2 following the manufacturer’s protocol (Clontech, Mountain View, CA). The titer of the rAd-MLC3f was adjusted to 1×10^12^ ifu/ml.

### Animals

The University of Minnesota’s Animal Care and Use Committee approved this protocol. Male Fischer-344 rats (10–12 months old) were purchased from the NIA colony (Indianapolis, IN) and housed in a temperature-controlled room (20±1°C) with a 12-hour light/12-hour dark cycle. Rats were acclimatized in the animal facility for at least one week and subsequently assigned to one of three groups: control (CON; n = 10), hindlimb unloading for 7 days (HU; n = 10), and HU for 7 days plus MLC3f cDNA treated (HUM; n = 10).

In a previous study we tested the influence of the adenovirus vector injections (with or without MLC3f cDNA) on skeletal muscle morphology and single fiber contractility [12]. We demonstrated that the adenovirus vector injections (rAD-empty vector, rAD-MLC3f vector) did not change single fiber contractility (force generation, Vo), did not increase muscle damage, did not increase inflammation, nor increase oxidative stress [12]. Because the adenovirus vector injections did not influence force generation in the previous study we selected to use this contractile parameter as the internal control for determining any negative impact of the adenovirus vector injections in the current study [12].

### Hindlimb unloading (HU)

Animals were HU for a one-week period to induce skeletal muscle dysfunction, including a reduction in contraction speed [14]. Briefly, using a tail harness tied to the proximal two-thirds of the tail the hindlimbs were elevated to a spinal orientation of 40°-45° above horizontal. The height of the unloading was vertically adjusted so the forelimbs remained in contact with the cage floor, thus allowing rats to move, and obtain food and water. All rats were cleaned to maintain their hygiene and monitored for cage activity, eating, and drinking two-times (morning/afternoon) daily during the HU period [15].

### The rAd-MLC3f injection into SM

**Fig 1:**
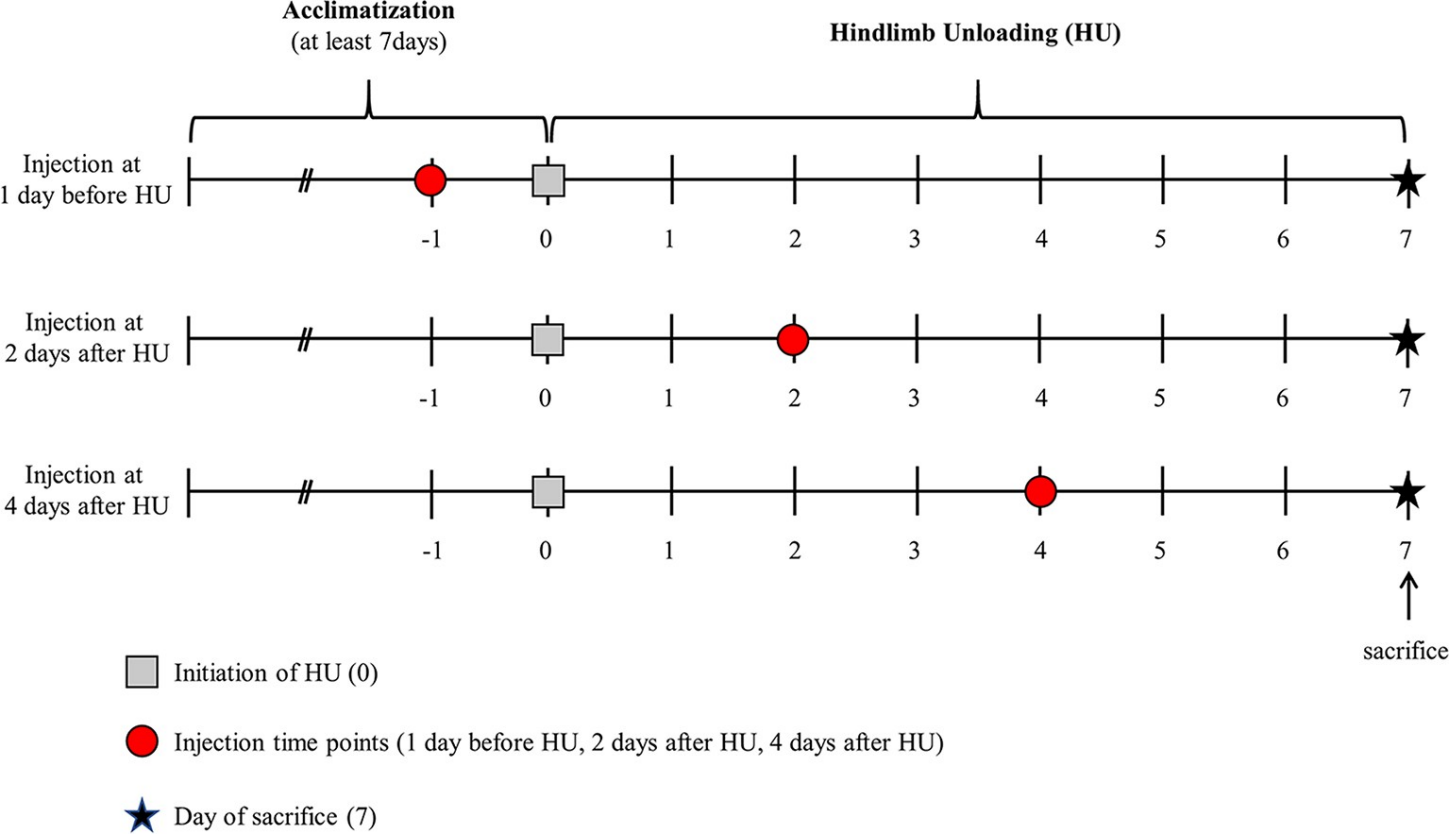
Experimental timeline to highlight day of rAd-MLC3f injection. The experimental timeline in days is divided into two phases [Acclimatization of at least 7 days and Hindlimb Unloading (HU) for 7 days]. The study evaluated three titers of the rAd-MLC3f 1×10^12^ ifu/ml, 2.5 x 10^11^, 3.75 x 10^11^ or 5 x 10^11^ ifu/ml and three injection timepoints. 250 μl total of the rAd-MLC3f was injected into five areas of the SM muscle of both hindlimbs and at one of the three-time points: one-day before HU (-1), two-days after HU (2), or four-days after HU (4).

To examine the effect of the rAd-MLC3f injection on MLC protein content and Vo in MHC type IIB single fibers, the animals in the HUM group were anesthetized with ketamine/xylazine (1:9 ratio) and removed from suspension. The rAd-MLC3f (250μl total) was carefully injected into five areas of SM muscle, which is predominantly composed of MHC type II fibers. Following the rAd-MLC3f delivery, the rats were allowed to recover from anesthesia and resuspended until the conclusion of the one-week period. A pilot study was performed to identify the optimal rAd-MLC3f injection titer and time point of injection during the HU period (Fig 1). The pilot study showed an injection of 3.75 x 10^11^–5 x 10^11^ ifu/ml at four days after the initiation of HU were the optimal experimental conditions to increase MLC3f expression. The supplemental materials contain the details of the pilot study including the other titers tested and the other time points of injection (S1–S3 Figs).

### Fiber bundle and permeabilized single fiber preparation

After the one-week experimental period, all rats were anesthetized using sodium pentobarbital (100mg/kg, ip injection). The SM muscles were rapidly dissected and trimmed free of excess fat and connective tissue. The muscles were weighed and then placed in cold relaxing solution (pCa = 9.0, pH = 7.0) containing 20mM imidazole, 7mM EGTA, 5.4mM MgCl_2_, 14.5mM creatine phosphate, 4.7mM ATP and CaCl_2_ to achieve pCa (-log[Ca^2+^]) 9.0 [16]. Because our previous investigation showed ~30% expression vector efficiency, which is a consistent result with previous research [17], we identified and isolated the infected fibers with a fluorescent microscope [12]. Subsequently, small fiber bundles (100–150 fibers per bundle) were prepared [12]. The bundles were placed in skinning solution containing 50% glycerol, 20mM imidazole (pH = 7.0), 125mM K-propionate, 2mM EGTA, 1mM MgCl_2_, 4mM ATP and stored at -20°C up to four weeks [15].

### Determination of unloaded shortening velocity (Vo)

On the day of the experiment, the single fiber was isolated from a bundle in relaxing solution, and each end of the fiber was connected with aluminum T-clips (Kem-Mil, CA). The fiber (~2mm long) was then transferred immediately to an experimental bath filled with relaxing solution (Permeabilized Fiber Apparatus Model 802B; Aurora Scientific, Aurora, Ontario, CAN) and mounted to a force transducer (400A; Aurora Scientific, CAN) and a high speed length controller (312C; Aurora Scientific, CAN) as described previously [12]. After confirming the fiber showed no damage the sarcomere length was adjusted at 2.4–2.5μm and fiber length (Lo) was determined. The fiber diameter was determined by measuring three different locations along the length of the fiber as previously described [12].

To determine the effect of HU and rAd-MLC3f injection on Vo, we utilized the Slack-test technique as previously described [12]. After the baseline force of a single muscle fiber was set to zero in relaxing solution, the fiber was then quickly moved to activating solution (pCa = 4.5, pH = 7.0; 20mM imidazole, 7mM EGTA, 5.4mM MgCl_2_, 14.5mM creatine phosphate, 4.7mM ATP and CaCl_2_ to achieve pCa (-log[Ca^2+^] 4.5) to induce force generation. Isometric maximal force (Po, μN) was measured at the point where the force output reached a maximum. The Slack test was initiated with the introduction of four to five slack distances between 10%-18% of Lo. When the fiber was slacked a specific distance the force generation dropped to zero initially and after a specific time interval force regeneration occurred. The time interval between the slack initiation and force regeneration was measured directly by custom software program (ASI Model 600A, Aurora Scientific, Ontario, CAN). The slope of the regression line (r^2^>0.98) was reported as Vo (fiber length per second; FL^-1^) after being normalized to fiber length (FL). The collected data from the force transducer and high-speed length controller were analyzed with a custom software program (ASI Model 600A, Aurora Scientific, Ontario, CAN). All experiments were conducted at 15°C.

### Determination of MHC and MLC compositions

**Fig 2:**
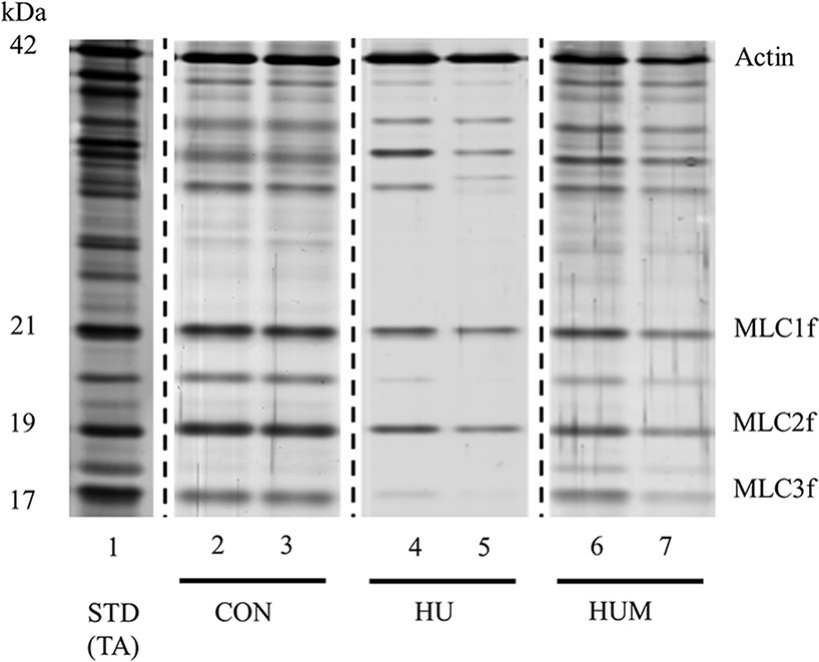
Representative MLC isoforms of single fibers in CON, HU, and HUM groups. The MLC isoforms within each individual skeletal muscle fiber were detected by 12% SDS-PAGE and silver staining technique. Each lane contains a single skeletal muscle fiber (lanes 2, 3 for CON, 4, 5 for HU, and 6, 7 for HUM). The ratio of MLC3f/MLC1f, MLC3f/MLC2f, and MLC3f/(MLC1f+MLC3f) was calculated by determining the relative content of MLC1f, MLC2f, and MLC3f with densitometry. Tibialis anterior (TA) muscle was used as a protein standard (STD), lane 1. The representative image was selected from control (CON), hindlimb unloading (HU), and HU for 7 days plus rAd-MLC3f injections (HUM; four-days after HU with 5 x 10^11^ ifu/ml). The dashed lines between the CON, HU, and HUM groups indicate that the gel lanes were taken from three separate gels.

After the single muscle fiber physiology experiments were completed, the MHC and MLC compositions of each fiber were determined by sodium dodecyl sulfate (SDS)-polyacrylamide gel electrophoresis (PAGE) and a custom silver staining method (Fig 2) [18]. Briefly, the fiber was solubilized in 50μl of 1% SDS sample buffer (24mM EDTA, 60mM Tris, 5% ß-mercaptoethanol, 2mg/ml bromophenol blue, 15% glycerol, 1% SDS; pH 6.8), and stored at -80°C. Subsequently, 10μl of each sample was loaded on gels consisting of a 4% acrylamide stacking gel and either a 5% separating gel for MHC or 12% separating gel for the MLC. The gels were washed, silver-stained, and scanned using molecular imaging system (GS-800; Bio-Rad, Hercules, CA). The reliability of our densitometry measurements (R^2^>0.99) was confirmed by a protein concentration curve and test-retest measure as previously described [12]. In the current study we report data (force generation, Vo and MLC relative content) from single fibers expressing the MHC type IIB isoform only (identified on the 5% SDS-PAGE gels). The relative contents of two essential MLC1f and MLC3f isoforms, and one regulatory MLC2f isoform were determined from the densitometric analysis using Image J software program as previously described [10]. The ratios of MLC3f/MLC1f, MLC3f/MLC2f, and MLC3f/(MLC1f+MLC3f) were also calculated as a preferable index of the ratio between essential and regulatory light chains in MHC type IIB muscle fibers [10]. In this study we determined Vo and MHC isoform content in a total of 316 single skeletal fibers. 134 fibers of the total 316 fibers tested contained pure MHC type IIB isoform. The pure MHC type IIB fibers were further evaluated for MLC isoform expression.

## Statistical analysis

One-way ANOVA was used to determine the mean differences in fiber size, force generation, MLC protein content and Vo among CON, HU, and HUM, followed by a Tukey’s post-hoc test when appropriate. To examine the relationship between the %MLC3f protein content and Vo in MHC type IIB fibers we performed a simple linear regression analysis. SPSS software (Version 24.0) was used for statistical analysis and statistics were expressed as mean ± SEM with significance at p-value < 0.05.

## Results

### rAd-MLC3f injection under conditions of HU does not cause further decreases in force generation

Table 1 summarizes the contractile characteristics for single fiber diameter, force generating capacity, and specific force in MHC type IIB fibers. The force generating capacity (Po) and the force generating capacity normalized to CSA (Po/CSA) were significantly reduced in the single MHC type IIB fibers from the HU and HUM experimental rats compared to the control rats. The force generating capacity of the single fibers from the HU rats receiving the rAd-MLC3f injection was not significantly different from the individual fibers from the HU rats not receiving the rAd-MLC3f injection. Taken together, the data indicated that the rAd-MLC3f injection does not cause further damage.

**Table 1:** Contractile characteristics of single MHC type IIB fibers.

	Variables		
Group	Diameter (μm)	Po (μN)	Po/CSA (kN/m^2^)
CON (57)	98.52±2.89	741.45±44.24	97.58±4.25
HU (44)	85.73±2.62*	379.09±23.77*	65.05±2.71*
HUM (33)	80.94±2.52*	443.27±38.73*	77.75±4.31*

Fiber diameter, maximal isometric force (Po), and specific tension (Po/CSA) of single MHC type IIB fibers from control (CON), HU for 7 days (HU), and HU for 7 days plus rAd-MLC3f injections (HUM). The number of fibers is presented in parentheses.

* indicates a significant difference from CON group. Significance was set at P<0.05 and values are expressed as mean ± SEM.

#### The relative MLC3f protein content and Vo in MHC type IIB fibers are reduced following HU

Table 2 summarizes the effect of HU on the relative MLC isoforms protein content (MLC1f, MLC2f, MLC3f) and Vo in MHC type IIB fibers. With HU, a 29% reduction in Vo was found and the percent of MLC3f was significantly reduced by 25%. In contrast to the reduced relative content of MLC3f and MLC2f isoforms, the percent of MLC1f was significantly increased with HU by 20%. The ratios of MLC3f/MLC1f and MLC3f/(MLC1f + MLC3f) were determined to support changes in relative MLC isoform protein content. Indeed, the ratios were also significantly lower with HU, 47% (MLC3f/MLC1f, p = 0.004) and 34% [MLC3f/(MLC1f + MLC3f), p<0.001, S1 Fig]. Collectively, the data indicated that one-week of HU significantly reduced the relative MLC3f protein content and Vo in MHC type IIB single fibers.

**Table 2:** The relative myosin light chain (MLC) protein content and unloaded shortening velocity (Vo) of single MHC type IIB fibers from control (CON) and hindlimb unloading (HU) groups.

	Variables			
Group	Vo (fl/s)	MLC1f (%)	MLC2f (%)	MLC3f (%)
CON	3.22±0.14 (57)	36.39±1.48 (74)	51.61±1.42 (74)	12.00±0.60 (74)
HU	2.27±0.08* (45)	43.75±1.38* (44)	47.19±1.23* (44)	9.06±0.66* (44)

Vo was determined by the slack test and the relative MLC protein content of each fiber was determined by 12% SDS-PAGE. The number of fibers is presented in parentheses.

* indicates a significant difference from CON group. Significance was set at P<0.05 and values are expressed as mean ± SEM.

### Increasing MLC3f content with rAd-MLC3f injection rescues HU-induced decline in Vo in MHC type IIB fibers

**Fig 3:**
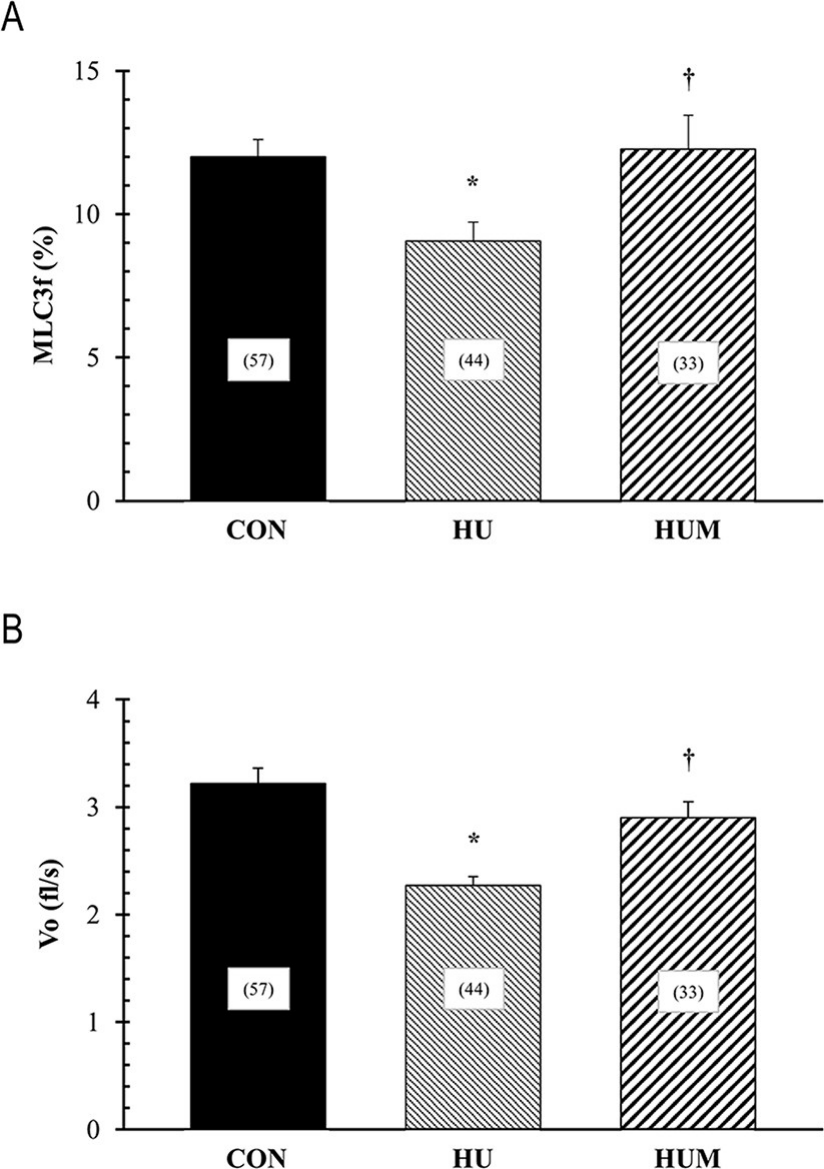
Percent of MLC3f protein content and Vo in single MHC type IIB fibers. The mean percent of MLC3f protein content (Panel A) and the mean unloaded shortening velocity (Vo, Panel B) of single MHC type IIB fibers from control (CON), HU for 7 days (HU), and HU for 7 days plus rAd-MLC3f injections (HUM). Vo was determined by the slack test and the percent of MLC3f protein content of each fiber was determined by 12% SDS-PAGE. The number of MHC type IIB fibers is shown in the boxes. * indicates a significant difference from CON group. † indicates a significant difference from HU group. Significance was set at P<0.05 and values are expressed as mean ± SEM.

In order to determine if increasing the relative MLC3f protein content results in a faster contraction speed in single fibers, we compared the relative MLC3f protein content and Vo of single MHC type IIB fibers from HU rats receiving the rAd-MLC3f injection (HUM) to HU rats without injection. Fig 3A shows a significant increase in MLC3f relative content in single MHC type IIB fibers in the HUM experimental group compared to the HU experimental group. Consistent with the increase in MLC3f relative content in single MHC type IIB fibers in the HUM experimental group Vo was significantly faster compared to the HU rats without injection (Fig 3B). In fact, the Vo in the HUM experimental group (2.90±0.15fl/s) was not significantly different than the Vo in the CON experimental group (3.22±0.14fl/s). Hence, the data indicate that the rAd-MLC3f injection (3.75 x 10^11^–5 x 10^11^ ifu/ml) at four-day after HU can effectively restore the HU-induced decline in Vo in MHC type IIB single fibers.

### The relationship between the relative MLC3f protein content and Vo is positive

**Fig 4:**
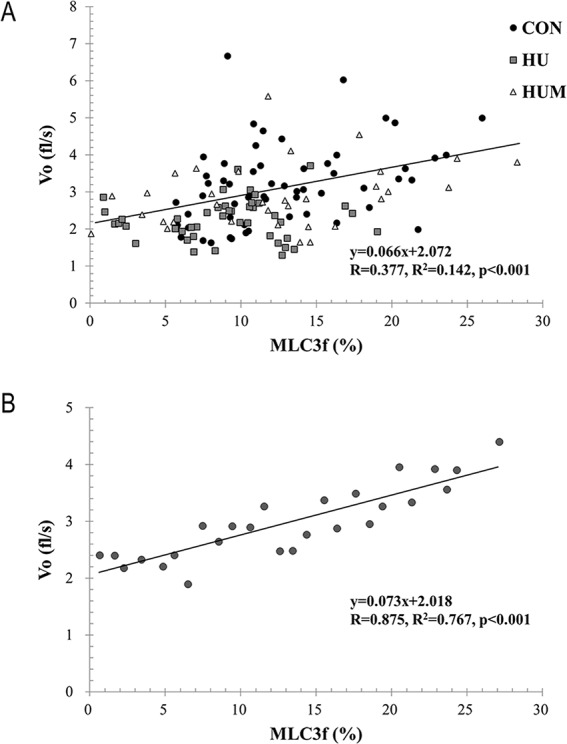
Relationship between Percent MLC3f content and Vo. The percent of MLC3f protein content and the corresponding Vo in each single MHC type IIB fiber investigated from CON, HU, and HUM (3.75 x 10^11^–5 x 10^11^ ifu/ml rAd-MLC3f injection) (Panel A). The relationship between percent of MLC3f and the corresponding Vo of single fibers where the fibers were grouped based on 1% intervals of MLC3f content and their respective mean Vo (Panel B). The simple linear regression analyses are presented.

Fig 4A plots the relative MLC3f content and Vo for single MHC type IIB fiber from Control, HU, and HUM (n = 134) experimental groups. A significant correlation between %MLC3f and Vo in MHC type IIB fibers (R = 0.377, p<0.001) was present. Fig 4B represents a plot where the fibers were grouped based on %MLC3f (1% intervals, ~14 fibers each interval, 1–27%) and their respective mean Vo. A positive relationship between %MLC3f protein content and Vo in MHC type IIB fibers was observed (R = 0.875, p<0.001).

## Discussion

Because MLC3f has the potential to regulate shortening velocity in MHC type IIB fibers, we hypothesized that increasing the relative MLC3f protein content via rAd-MLC3f injection would attenuate HU-induced decline in Vo. The major findings of this study include that the relative MLC3f content and Vo in MHC type IIB fibers are significantly reduced following one-week of HU, the administration of the rAd-MLC3f significantly attenuates the HU-induced decline in MLC3f content and Vo in MHC type IIB fibers, and there is a positive relationship between percent of MLC3f content and Vo. Collectively, increased MLC3f protein content, via rAd-MLC3f, may provide a potential protection against HU-induced decline in Vo in MHC type IIB single muscle fibers.

### Essential MLCs and Vo in MHC type IIB fibers

The MHC isoform is the key player in regulating contractile velocity, with single fibers composed of MHC type IIB isoform contracting four times faster than single fibers composed of MHC type I isoform [2]. The contractile velocity of single fibers with MHC type IIB isoforms shows a large variability or continuum, with studies reporting 1.5 to 6.7fl/s, a 4.5 fold difference [2,12]. This continuum of contractile velocities within MHC type IIB fibers is associated with the MLC isoform composition because the amino acid sequence between the essential MLCs isoforms, MLC1f and MLC3f are different [1,6]. Indeed, the forty-five amino acid N-terminal extension of essential MLC1f causes an unfavorable interaction with the negatively charged C-terminus of actin, but this interaction is lessened in the essential MLC3f isoform that has a relatively shorter amino acid sequence [6]. At the cross-bridge the interactions between MLC1f and actin increase the lifetime of the attached state of the myosin heads, delay their dissociation, resulting in a slower contraction [6]. In contrast, the interactions between MLC3f isoform and actin reduce the actomyosin attachment/detachment rate. Therefore, fibers with more MLC3f isoform contract faster than the fibers with more MLC1f [1]. In contrast to the contractile property of speed, both the ATPase activity and the stretch-induced force transients (single fibers, actomyosin in solution) are not associated with the relative content of MLC3f and MLC1f isoforms [3,19–21].

### The relative MLC3f content and Vo decrease in MHC type IIB fibers with HU

Disuse or unloading is known to be associated with changes in muscle cellular function and properties. In MHC type I fibers from the soleus, there is significant atrophy and increases in contraction velocity [22]. The reported mechanism for the elevated speed is related to sarcomere structure, where the myosin/actin lattice spacing is altered, resulting in favorable cross-bridge kinetics [23]. Most likely the altered lattice spacing is due to preferential degradation of actin compared to myosin during the process of atrophy involved with non-weight bearing conditions [24]. In addition to the electron microscopy images revealing lattice spacing, alterations in the myosin:actin protein ratio following conditions of non-weight bearing provide further evidence for this preferential degradation [15,25,26]. An increase in MLC3f content may also contribute to the faster contraction speed in the MHC type I fibers from the soleus muscle [9].

In contrast to the MHC type I fibers, a decrease in contraction velocity in MHC type IIB fibers with non-weight bearing is observed. This reduction in velocity is reported in several models of non-weight bearing (e.g., bedrest, hindlimb unloading, spaceflight) and in several species (non-human primate, rat) [10,11,23,27]. The decrease in velocity is present not only in single skeletal muscle fibers but also in the type IIB myosin motor (isolated myosin) and the sub-fragment, heavy meromyosin (HMM) [11]. The contractility characteristics of the type IIB myosin motor and HMM are investigated using the *in vitro* motility assay [11]. Maffei et al. [11] investigated three potential underlying mechanisms for the reduced velocity, MLC isoform composition, myosin oxidation, and MLC2 phosphorylation [10,11]. The evidence from the HMM studies indicates myosin oxidation is not involved. The whole muscle evidence suggests a potential involvement of phosphorylation of MLC2. Although Maffei et al. [11] did not report any change in MLC1f and MLC3f at the whole muscle level, the relative content of the MLC1f and MLC3f within single fibers, type IIB myosin motor and the sub-fragment, HMM is not reported; hence, this underlying mechanism cannot be eliminated. Most likely, the detection of the MLCs within the single molecule is not possible with current technology.

One previous investigation reported alterations in MLC isoform expression levels at two, three, and four weeks of non-weight bearing and concomitant decreases in contractile velocity in MHC type IIB fibers of 26–38% were observed [10]. In fact, MLC3f protein content decreased and there was an increase of MLC1f isoform by 17% at two weeks of HU. There was a correlation between the relative content of MLC3f isoform and contraction speed. Interestingly, the velocity was not further reduced in the MHC type IIB single fibers with longer periods of non-weight bearing (3, 4 weeks). Consistent with the previous work, the current study shows the rapid decline in contraction speed at 1 week, a 29% reduction with the concomitant decrease in MLC3f (and an increase in MLC1f). Collectively, the data indicate the reduction in contractile speed occurs rapidly and in the early phases of non-weight bearing.

HU or non-weight bearing is associated with severe muscle atrophy, changes in protein turnover with increased protein degradation and decreased protein synthesis processes. Indeed, the process of muscle atrophy is complex, is initiated immediately upon removal of weight bearing, and the proteins within the myofibril disassemble in a selective manner [28]. Important to the current study and the observed changes in the relative contents of the two MLC isoforms (MLC1f and MLC3f), Cohen and colleagues [28] demonstrate that the essential MLCs are degraded in the early phase of atrophy process by MuRF1-dependant ubiquitination and these ubiquitinated proteins disturb muscle contractile structure and function (e.g., 3 days of denervation). In fact, this research team suggests that the loss of MLCs (both essential and regulatory) and the MyBP-C (myosin binding protein C) during the initial steps of thick filament disassembly (e.g., early phase) may enhance the susceptibility of other myofibrillar components to degradation.

The work of Kirschbaum and colleagues [29] further demonstrate a coordination of MLC1f and MLC3f degradation in a chronic stimulation model, whereby MLC3f is more rapidly degraded compared to MLC1f. To support the enhanced degradation processes, key components of the proteasome, calpain, and cathepsin pathways are upregulated (mRNA and protein) whereas the key components of the synthesis pathways are downregulated with varied models of muscle atrophy. Collectively, selective degradation of MLC3f contributes to the altered relative content of the MLC isoforms.

### Strategies to attenuate declines in contractile velocity

One strategy to attenuate the HU-induced decline in contractile speed in MHC type IIB fibers is to increase the MLC3f content via rAd-MLC3f. This strategy has been successful in an aging model where there is a slowing of contraction in MHC type II fibers [12]. Specifically, this investigation not only revealed a correlation between speed and the essential MLC isoform content in single MHC type IIB fibers but demonstrated an age-related reduction in contraction speed and expression levels of MLC3f [12]. Because the rAd-MLC3f transduction in the SM muscle was successful in rescuing age-related slowing of contraction without increasing cellular inflammation and contractile protein damage, we hypothesized it would be possible to use this strategy to attenuate the HU-induced decline.

The primary goal of the current study was to restore the HU-induced slowing of muscle contraction by increasing the relative content of MLC3f. Indeed, an overall increase in relative MLC3f content in MHC type IIB fibers was observed after rAd-MLC3f injection. The increased content to 12.26% from 9.06% via rAd-MLC3f gene transfer to SM muscle significantly offset HU-induced decline in Vo from 2.27fl/s to 2.90fl/s. This finding further substantiates the important role of the MLCs in modulating contraction speed.

Although the essential lights chains (MLC1f and MLC3f) are not required for contraction speed [30], when present there is a linear relationship between MLC isoform content and contraction speed using various technologies [2,5,12]. Notably, a small change in the MLC isoform content in single fiber preparations has a significant impact. For instance, a strong linear correlation coefficient between velocity and relative amount of MLC3 (R = 0.858, p<0.001) is reported [5]. In the current study, a similar linear relationship (R = 0.875, p<0.001) is observed when all the individual fibers are analyzed together from the three experimental groups, with the impact of increasing MLC3f content from 0% to 20% results in a 72% increase in speed (velocity from 2.02fl/s to 3.48fl/s). Taken together, this provides a molecular mechanism for the physiological deterioration in velocity in type IIB fibers following non-weight bearing and the benefits of rAd-MLC3f to restore function.

One caveat is noted. In Fig 4 (panels A and B) we demonstrate a significant relationship between %MLC3f and Vo when we analyzed all the single MHC type IIB fibers from the three experimental groups (CON, HU, HUM). However, if we analyze the relationship between %MLC3f and Vo in the MHC type IIB fibers from the hindlimb unloaded rats separately (the HU experimental group only), the linear relationship is not present (R = 0.066, p = 0.669, solid square symbols). The underlying mechanisms responsible for this loss are unknown. Potential mechanisms include protein damage or changes in the phosphorylation state of the regulatory light chains [11,31,32], both associated with the muscle degradation processes [11]. Therefore, further studies are required to elucidate exact roles of protein degradation, post-translational modification, and the regulatory proteins (particularly, MLC2f phosphorylation) on muscle activation kinetics in single muscle fibers.

In summary, HU results in a decrease in Vo in MHC type IIB fibers and the decrease in Vo is in part due to a decrease in the relative MLC3f content and an increase in the relative MLC1f content. Our data provide the evidence that HU-induced reduction in contraction speed may be attenuated by the gene delivery of MLC3f, which may eventually delay the rapid physical decline and limited quality of life.

## Supporting information

S1 Fig **Percent of MLC3f/MLC1f (A), MLC3f/MLC2f (B), and MLC3f/(MLC1f+MLC3f) (C) in single MHC type IIB fibers from SM muscles following HU (7days) and a 2.50 x 10**^**11**^ **ifu/ml rAd-MLC3f injection.** Experimental groups include: CON, (n = 74); HU, (n = 44); HUM-1D, the rAd-MLC3f injection was administered one-day before the initiation of HU (eight-day before sacrifice) (n = 14); HUM+2D, the rAd-MLC3f injection was administered two-days after the initiation of HU (five-days before sacrifice) (n = 14); HUM+4D, the rAd-MLC3f injection was administered four-days after the initiation of HU (three-days before sacrifice) (n = 10). * indicates a significant difference with CON group. Significance was set at P<0.05 and values are expressed as mean ± SEM. Panel A, B, and C indicate the percent of MLC3f/MLC1f, MLC3f/MLC2f, and MLC3f/(MLC1f+MLC3f), respectively.
(TIF)

S2 Fig **Percent of MLC3f/MLC1f (A), MLC3f/MLC2f (B), and MLC3f/(MLC1f+MLC3f) (C) in single MHC type IIB fibers from SM muscles following HU (7days) and a 3.75 x 10**^**11**^ **ifu/ml rAd-MLC3f injection.** Experimental groups include: CON, (n = 74); HU, (n = 44); HUM-1D, the rAd-MLC3f injection was administered one-day before the initiation of HU (eight-day before sacrifice) (n = 16); HUM+2D, the rAd-MLC3f injection was administered two-days after the initiation of HU (five-days before sacrifice) (n = 19); HUM+4D, the rAd-MLC3f injection was administered four-days after the initiation of HU (three-days before sacrifice) (n = 18). * indicates a significant difference with CON group. † indicates a significant difference from HU group. Significance was set at P<0.05 and values are expressed as mean ± SEM. Panel A, B, and C indicate the percent of MLC3f/MLC1f, MLC3f/MLC2f, and MLC3f/(MLC1f+MLC3f), respectively.
(TIF)

S3 Fig **Percent of MLC3f/MLC1f (A), MLC3f/MLC2f (B), and MLC3f/(MLC1f+MLC3f) (C) in single MHC type IIB fibers from SM muscles following HU (7days) and a 5 x 10**^**11**^ **ifu/ml rAd-MLC3f injection.** Experimental groups include: CON, (n = 74); HU, (n = 44); HUM-1D, the rAd-MLC3f injection was administered one-day before the initiation of HU (eight-day before sacrifice) (n = 10); HUM+2D, the rAd-MLC3f injection was administered two-days after the initiation of HU (five-days before sacrifice) (n = 11); HUM+4D, the rAd-MLC3f injection was administered four-days after the initiation of HU (three-days before sacrifice) (n = 15). * indicates a significant difference with CON group. † indicates a significant difference from HU group. Significance was set at P<0.05 and values are expressed as mean ± SEM. Panel A, B, and C indicate the percent of MLC3f/MLC1f, MLC3f/MLC2f, and MLC3f/(MLC1f+MLC3f), respectively.
(TIF)
