# Supplementary figures and images for: Increasing myosin light chain 3f (MLC3f) protects against a decline in contractile velocity
Source: PLoS One. 2019 Apr 9;14(4):e0214982. doi: 10.1371/journal.pone.0214982 (PMC6456215; doi:10.1371/journal.pone.0214982)

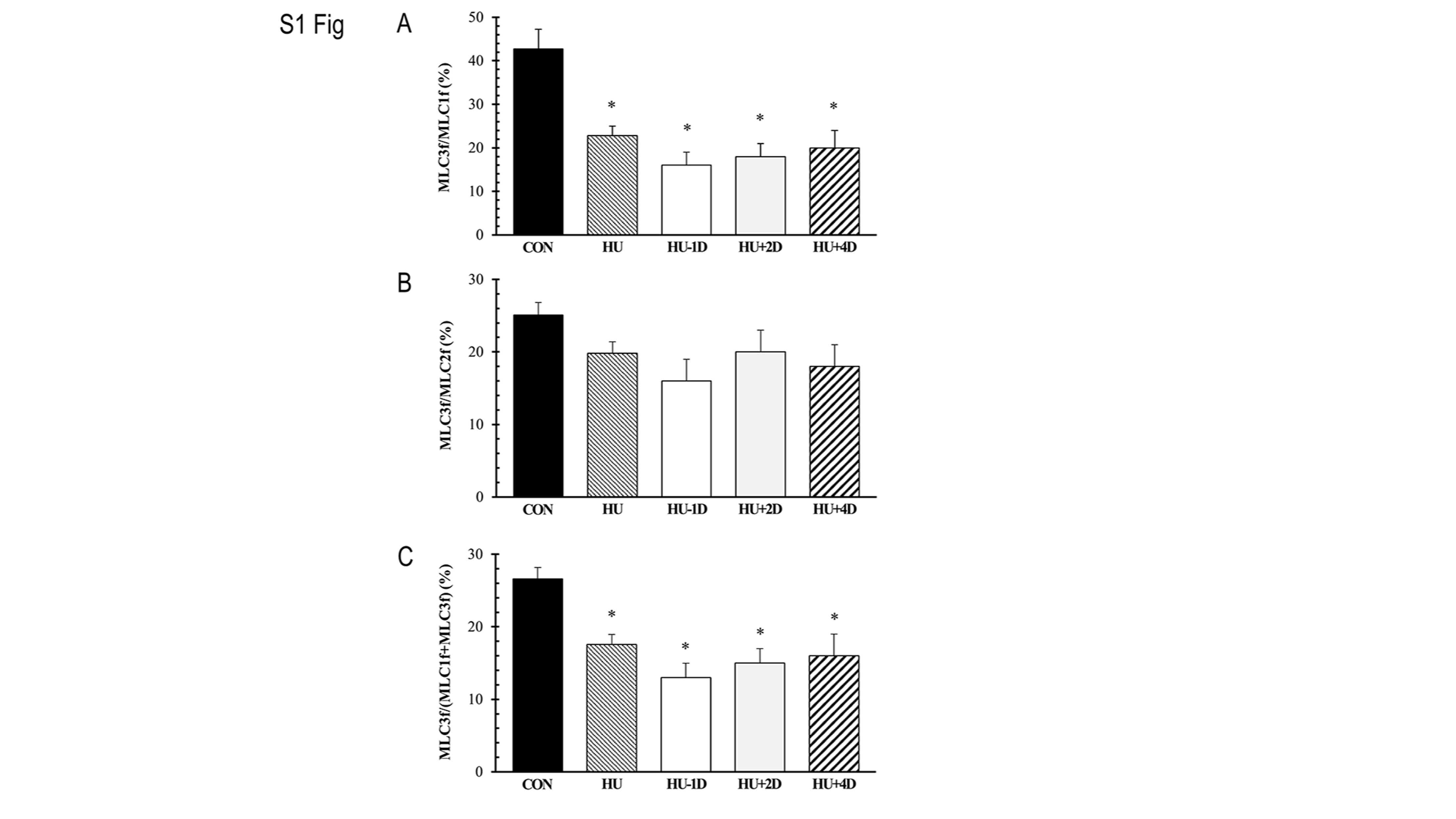

Supplement: S1 Fig — Percent of MLC3f/MLC1f (A), MLC3f/MLC2f (B), and MLC3f/(MLC1f+MLC3f) (C) in single MHC type IIB fibers from SM muscles following HU (7days) and a 2.50 x 10^11^ ifu/ml rAd-MLC3f injection. Experimental groups include: CON, (n = 74); HU, (n = 44); HUM-1D, the rAd-MLC3f injection was administered one-day before the initiation of HU (eight-day before sacrifice) (n = 14); HUM+2D, the rAd-MLC3f injection was administered two-days after the initiation of HU (five-days before sacrifice) (n = 14); HUM+4D, the rAd-MLC3f injection was administered four-days after the initiation of HU (three-days before sacrifice) (n = 10). * indicates a significant difference with CON group. Significance was set at P<0.05 and values are expressed as mean ± SEM. Panel A, B, and C indicate the percent of MLC3f/MLC1f, MLC3f/MLC2f, and MLC3f/(MLC1f+MLC3f), respectively. (TIF) [file pone.0214982.s001.tif]

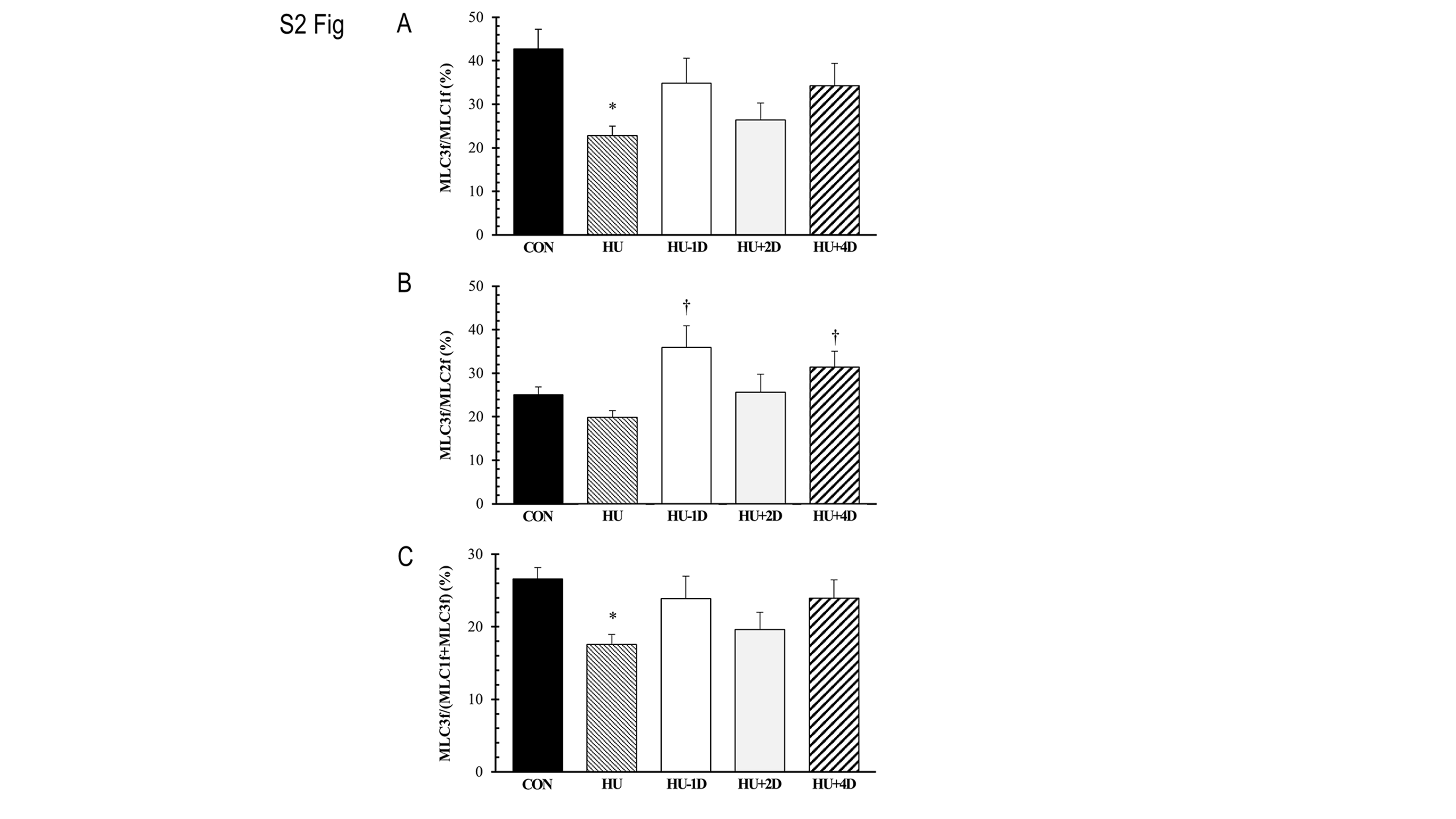

Supplement: S2 Fig — Percent of MLC3f/MLC1f (A), MLC3f/MLC2f (B), and MLC3f/(MLC1f+MLC3f) (C) in single MHC type IIB fibers from SM muscles following HU (7days) and a 3.75 x 10^11^ ifu/ml rAd-MLC3f injection. Experimental groups include: CON, (n = 74); HU, (n = 44); HUM-1D, the rAd-MLC3f injection was administered one-day before the initiation of HU (eight-day before sacrifice) (n = 16); HUM+2D, the rAd-MLC3f injection was administered two-days after the initiation of HU (five-days before sacrifice) (n = 19); HUM+4D, the rAd-MLC3f injection was administered four-days after the initiation of HU (three-days before sacrifice) (n = 18). * indicates a significant difference with CON group. † indicates a significant difference from HU group. Significance was set at P<0.05 and values are expressed as mean ± SEM. Panel A, B, and C indicate the percent of MLC3f/MLC1f, MLC3f/MLC2f, and MLC3f/(MLC1f+MLC3f), respectively. (TIF) [file pone.0214982.s002.tif]

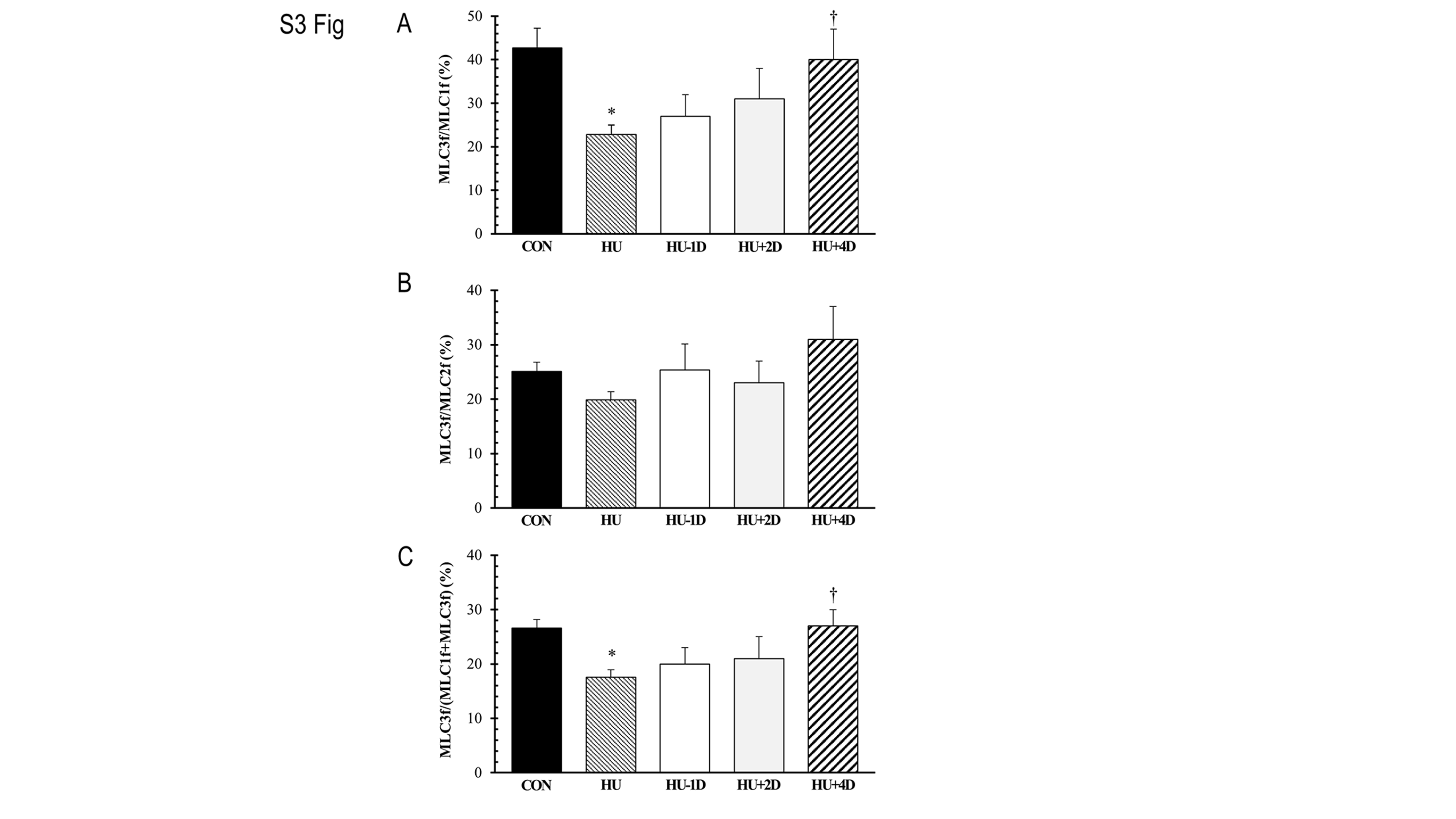

Supplement: S3 Fig — Percent of MLC3f/MLC1f (A), MLC3f/MLC2f (B), and MLC3f/(MLC1f+MLC3f) (C) in single MHC type IIB fibers from SM muscles following HU (7days) and a 5 x 10^11^ ifu/ml rAd-MLC3f injection. Experimental groups include: CON, (n = 74); HU, (n = 44); HUM-1D, the rAd-MLC3f injection was administered one-day before the initiation of HU (eight-day before sacrifice) (n = 10); HUM+2D, the rAd-MLC3f injection was administered two-days after the initiation of HU (five-days before sacrifice) (n = 11); HUM+4D, the rAd-MLC3f injection was administered four-days after the initiation of HU (three-days before sacrifice) (n = 15). * indicates a significant difference with CON group. † indicates a significant difference from HU group. Significance was set at P<0.05 and values are expressed as mean ± SEM. Panel A, B, and C indicate the percent of MLC3f/MLC1f, MLC3f/MLC2f, and MLC3f/(MLC1f+MLC3f), respectively. (TIF) [file pone.0214982.s003.tif]
